# ATPase Activity of Bacillus subtilis RecA Affects the Dynamic Formation of RecA Filaments at DNA Double Strand Breaks

**DOI:** 10.1128/msphere.00412-22

**Published:** 2022-11-02

**Authors:** Rogelio Hernández-Tamayo, Niklas Steube, Thomas Heimerl, Georg K. A. Hochberg, Peter L. Graumann

**Affiliations:** a Center for Synthetic Microbiology (SYNMIKRO), Marburg, Germany; b Department of Chemistry, Philipps-Universität Marburg, Marburg, Germany; c Max Planck Institute for Terrestrial Microbiology, Marburg, Germany; d Department of Biology, Philipps-Universität Marburg, Marburg, Germany; University of Iowa

**Keywords:** homologous recombination, RecA, DNA break repair, *Bacillus subtilis*, DNA repair, double strand break repair, single molecule tracking

## Abstract

RecA plays a central role in DNA repair and is a main actor involved in homologous recombination (HR). *In vivo*, RecA forms filamentous structures termed “threads,” which are essential for HR, but whose nature is still ill defined. We show that RecA from Bacillus subtilis having lower ATP binding activity can still form nucleoprotein filaments *in vitro*, features lower dsDNA binding activity, but still retains most of wild type RecA activity *in vivo*. Contrarily, loss of ATPase activity strongly reduced formation of nucleoprotein filaments *in vitro*, and effectivity to repair double strand breaks (DSBs) *in vivo.* In the presence of wild type RecA protein, additionally expressed RecA with lowered ATPbinding activity only moderately affected RecA dynamics, while loss of ATPase activity leads to a large reduction of the formation of threads, as well as of their dynamic changes observed in a seconds-scale. Single molecule tracking of RecA revealed incorporation of freely diffusing and nonspecifically DNA-bound molecules into threads upon induction of a single DSB. This change of dynamics was highly perturbed in the absence of ATPase activity, revealing that filamentous forms of RecA as well as their dynamics depend on ATPase activity. Based on the idea that ATPase activity of RecA is most important for DNA strand exchange activity, our data suggest that extension and retraction of threads due is to many local strand invasion events during the search for sequences homologous to the induced DNA break site.

**IMPORTANCE** Single-strand (ss) DNA binding ATPase RecA is the central recombinase in homologous recombination, and therefore essential for DNA repair pathways involving DNA strand exchange reactions. In several bacterial, RecA forms filamentous structures along the long axis of cells after induction of double strand breaks (DSBs) in the chromosome. These striking assemblies likely reflect RecA/ssDNA nucleoprotein filaments, which can extend and remodel within a time frame of few minutes. We show that ATPase activity of RecA is pivotal for these dynamic rearrangements, which include recruitment of freely diffusing molecules into low-mobile molecules within filaments. Our data suggest that ssDNA binding- and unbinding reactions are at the heart of RecA dynamics that power the dynamics of subcellular filamentous assemblies, leading to strand exchange reactions over a distance of several micrometers.

## INTRODUCTION

All cells possess an intricately regulated response to DNA damage. Bacteria have evolved an extensive regulatory network called the SOS response to control the synthesis of factors that protect and repair the genome. Processes coordinately regulated within the SOS response include error-free DNA repair (1), error-prone lesion bypass (2, 3), inhibition of cell division (4), and homologous recombination (HR) (5, 6).

HR is a mechanism used by all organisms to repair DNA lesions such as double stranded breaks (DSBs) and collapsed replication forks (3, 7, 8). DSBs can be repaired through 2 principally different processes: error-prone direct end joining, or recombination with the intact DNA duplex of the sister chromosome (9). To carry out its central function in HR, RecA must assemble into helical nucleoprotein filaments on the ssDNA tracts formed at sites of DSBs or stalled replication forks. Although RecA binds rapidly to ssDNA in purified systems, its ability to bind tracts of ssDNA *in vivo* requires one of 2 pathways that allow RecA to outcompete the high affinity ssDNA binding protein SSB. Initially, during presynapsis, DNA ends are primed for loading of the central homologous recombination protein RecA (Rad51 in eukaryotes) by the MRX (Mre11–Rad50–Xrs2) complex in eukaryotic cells or by RecBCD, RecF, RecO, and RecR proteins in bacteria (9–13). During synapsis, RecA sets up strand exchange by introducing a single DNA strand from the break site into the intact homologous sister chromosome, and vice versa. Finally, during post synapsis, RecA-mediated three-way junctions are converted into true crossovers (or Holliday junctions) through the action of proteins such as the RecG helicase, and branch migration and resolution of Holliday junctions are achieved through the action of the RuvABC complex in bacteria (14). RecA is a DNA-dependent ATPase (7, 15, 16). The ATP bound form of Escherichia coli RecA has a higher affinity for ssDNA and dsDNA than does the ADP-bound form (17). Thus, ATP hydrolysis converts a high affinity DNA binding form, RecA-ATP, to a low affinity form RecA-ADP, thereby supporting an ATP hydrolysis-dependent dynamic cycle of DNA binding and dissociation.

This difference in affinity, combined with the protein’s DNA-dependent ATPase activity, results in a dynamic DNA binding cycle: RecA-ATP cooperatively binds DNA to form helical nucleoprotein filaments; ATP hydrolysis throughout the filament converts RecA-ATP to RecA-ADP; and RecA-ADP protomers dissociate from DNA. Filament extension on ssDNA is less cooperative, possibly resulting in the formation of short filament patches as observed for RAD51 (15, 17). The 3′ end is typically favored for addition of RecA monomers and disassembly occurs from the 5′ end resulting in a net 5′ to 3′ assembly direction on ssDNA (18, 19). Due to the 5′ to 3′ directionality, the 3′ end is more likely to be covered with RecA resulting in more efficient pairing reactions at the 3′ end *in vitro* (20, 21). Resolved structures of RecA complexed with ssDNA indicate that ATP binding and ATP hydrolysis indeed mediate the binding and release of RecA from DNA through allosteric coupling (22). ATP hydrolysis is, however, not the only driving factor for dissociation, as the mechanical interactions of a recombinase filament with its stretched DNA substrates also facilitate disassembly (23). Although RecA is a DNA-dependent ATPase, its homology search and strand exchange activities are largely independent of its ATPase activity (24).

Proteins that have ATP hydrolytic activity typically have 2 well-defined motifs called the Walker A and B motifs (25). Researchers wishing to test the importance of ATP binding and hydrolysis in biological roles for these proteins create 2 types of mutations in the Walker A or P-loop motif. Both mutate the highly conserved lysine residue. A conservative change of the lysine residue to an arginine in many cases creates a protein that can still bind ATP but is no longer able to hydrolyze it (26–28). The second type replaces the lysine with an alanine. Here, the protein is commonly found to have greatly reduced ability to bind ATP (29–32). Despite RecA having been analyzed in depth in the Gram-positive model bacterium Bacillus subtilis *in vivo* and *in vitro*, loss of ATP binding or of ATPase activity have not yet been tested *in vivo*.

Interestingly, when DNA modifications are induced that lead to the generation of double strand breaks, or if defined DSBs are induced in the chromosome of bacteria, RecA assembles into filament-like structures that have been termed “threads” (33). It has been suggested that filamentous RecA structures are ssDNA RecA filaments that guide ssDNA from the break site toward the homologous DNA region that is generally located in the other cell half in B. subtilis cells (33), or to form protein filaments that guide a chromosome site containing a DSB toward the non-broken sister site in the other cell half (34). Because in general, bacteria rapidly segregate replicated regions on the chromosome into opposite cells half, sister loci are separate from each other, and are moved together in E. coli or Caulobacter crescentus cells, when a DSB is induced in one site (34–36). RecA has been proposed to mediate repairing of separated sister loci, by providing a track for e.g., motor proteins moving DSB sites (34), or by providing directionality for the search of the sister site (36) that is found due to sufficient sequence homology. In B. subtilis cells grown to the state of competence, DNA is taken up at a single cell pole. From this site, RecA forms threads that have been shown to be essential for homologous recombination during transformation with externally acquired DNA (37). Filaments have been shown to be about 60 nm thin (36), while single RecA/ssDNA filaments are 3 to 4 nm wide, and to remodel within a time frame of minutes in several studies (33, 34, 36, 37). It has been proposed that they might perform a motor-like function in bringing together sites of DSBs and the homologous sister site, or to transport ssDNA from the DSB site to the homologous site within the other cell half. Especially, how filament dynamics are driven is still unclear, as well as their mode of action.

In this study, we addressed the question: if loss of ATP binding or ATPase activity affect properties of RecA *in vitro*, or thread dynamics *in vivo*. *RecA*_K70R_ and *recA*_K70A_ alleles were placed into the *recA* locus, and translational fusions to mVenus or sfGFP were generated. These strains were assayed for their ability to repair and recombine DNA, and to induce a DSB-response by forming dynamic RecA threads on the DNA. Dynamics of RecA were assayed by superresolution (SIM) light microscopy and at single molecule level *in vivo*, yielding insight into the role of RecA-ATPase activity for nucleating RecA filaments. In our work, we developed a probe that specifically visualizes and quantifies RecA structures on DNA using a single molecule tracking approach, and utilize it to provide a detailed timeline of RecA structural organization in living cells after DSBs. We show that biochemical properties of B. subtilis RecA differ from those reported for E. coli RecA with respect to changes in ATP binding or ATPase activity, and that the latter is crucial for filament dynamics *in vivo*. This finding has important implications for the conclusions that can be drawn from filament dynamics observed in live cell imaging.

## RESULTS

### Characterization of Walker A mutations in RecA *in vitro*.

**FIG 1:**
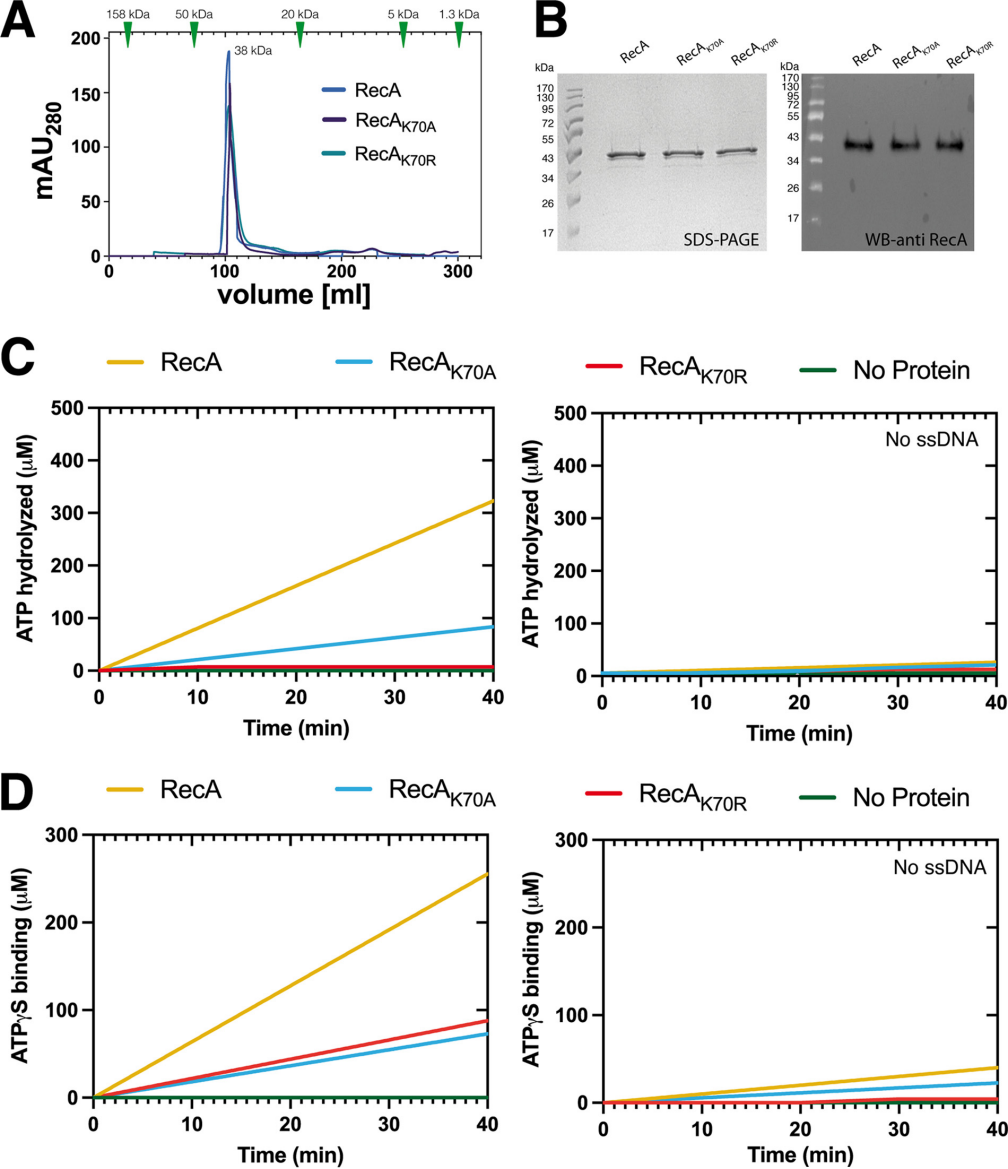
Biochemical characterization of RecA Walker A mutants. (A) Gel filtration (GF) of His Tag-RecA_WT_ and mutants after Nickel-Sepharose affinity chromatography and ensuing size exclusion chromatography. Size standards are shown with triangles in the upper part of the chromatogram. (B) SDS-PAGE and Western blot (WB) of fractions corresponding to elution fractions in GF fractions, for WB, proteins were probed using a 1:5000 dilution (rabbit-α-RecA) and secondary goat-α-rabbit-antibody (1:10000 dilution). (C) ATP hydrolysis assay as described in Material and Methods. (D) Kinetic analysis of ATP binding assay as described in Material and Methods.

RecA ATPase activity has been studied extensively *in vitro* (24, 38), but its requirements for recombination, DNA repair, and DSBs induction in the Gram-positive model bacterium B. subtilis have not yet been analyzed *in vivo*. Wild type RecA requires ATP binding for efficient loading onto ssDNA, and shows ATPase activity during DNA strand exchange, and during unloading from ssDNA filaments (39). A major aspect of our work was to determine if the formation of dynamic thread-structures requires ATP binding or ATP hydrolysis *in vivo*. To this end, we purified wild type RecA, and 2 Walker A mutant forms expected to abolish ATP binding (K70A) or to allow for ATP binding but prevent ATPase activity (K70R) as C-terminal hexa-histidine fusion proteins, via nickel-NTA affinity chromatography, and in a second step via size exclusion chromatography. Figure 1A shows that all 3 variants eluted in a single peak corresponding to RecA monomers. However high salt conditions (500 mM NaCl) that were required to remove residual, bound DNA from the overexpression host, may drive RecA into its monomeric form (see below). Figure 1B shows that all variants were obtained in high purity. For all 3 variants, we did not observe any ATPase activity in the absence of ssDNA (Salmon Sperm ssDNA), but activity of wild type RecA comparable to that of E. coli RecA (Fig. 1C), indicating that the C-terminal affinity purification tag of RecA does not interfere with its ATPase activity. Surprisingly, we found lower, but substantial activity for the RecA_K70A_ mutant, while there was no detectable activity for RecA_K70R_ (Fig. 1C). This is due to residual ATP binding for RecA_K70A_, which was about 3-fold lower compared to that of wild type RecA, in the presence of ssDNA. RecA_K70R_ mutant also showed about 3-fold reduced ATP binding activity, while in the absence of ssDNA, there was no binding activity, which for wild type RecA was also strongly reduced (Fig. 1D).

Thus, while the lysine to arginine substitution behaved roughly to our expectation, losing ATPase activity but not completely ATP binding, the alanine exchange did not result in a complete loss of ATP binding, but to a strong reduction and, thus, also to decreased ATPase activity. These findings put us into the situation of addressing the question if loss of ATPase activity leads to changes in RecA activity and/or formation of threads during DNA repair.

### Loss of ATPase activity affects nucleofilament formation *in vitro*.

**FIG 2:**
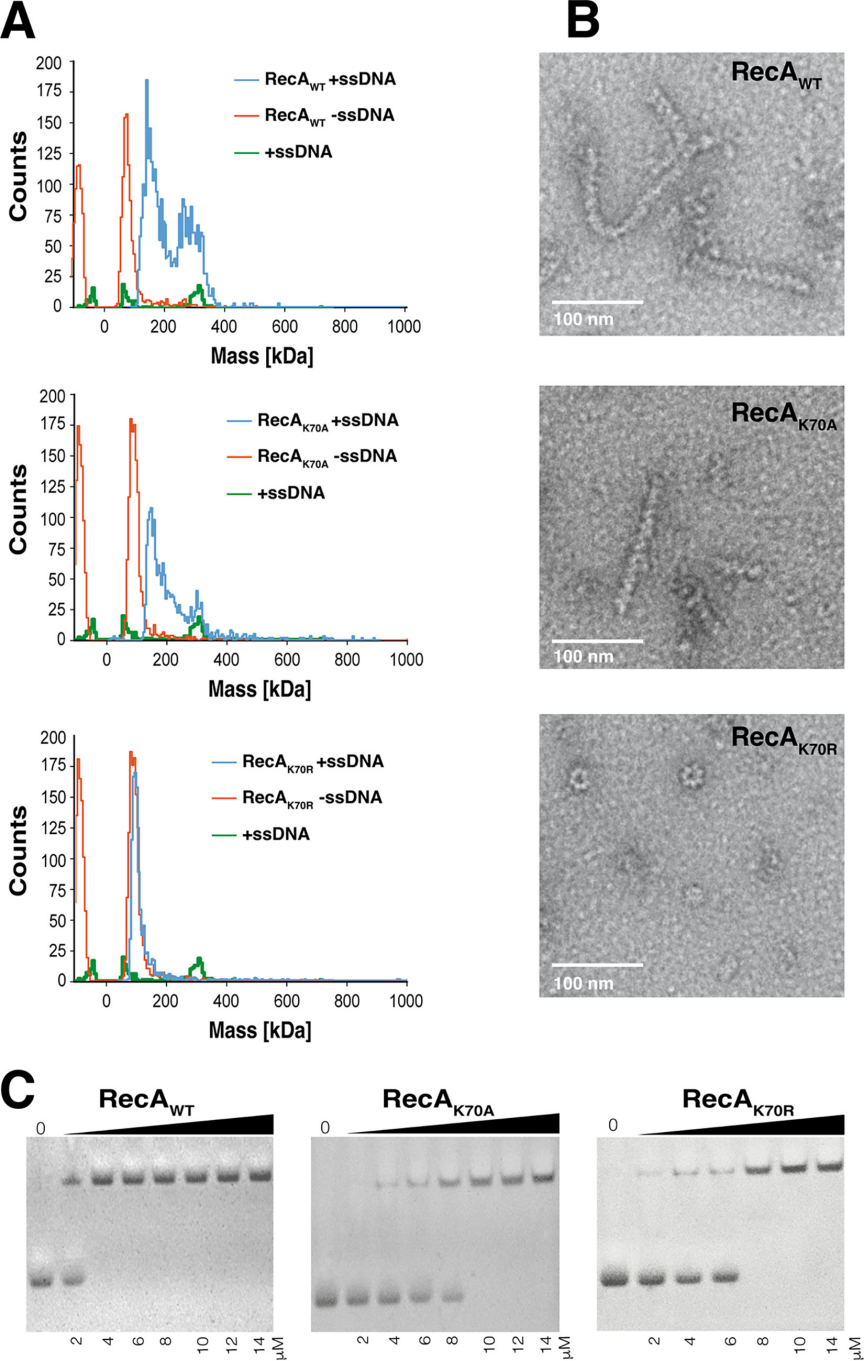
Filament formation of RecA and ATPase mutants in mass photometry and electron microscopy. (A) Molecular mass distribution histograms of RecA_WT_, RecA_K70A_ or RecA_K70R_ alone or incubated with ssDNA and ATP. (B) Electron micrographs show filament formation by negative staining of ssDNA and wild type RecA or Walker A mutants. (C) DNA binding of RecA, EMSAs showing RecA_WT_, RecA_K70A_ or RecA_K70R_ binding to dsDNA.

We wished to analyze the effects of Walker A mutations in B. subtilis RecA on the formation of nucleofilaments *in vitro*. We therefore analyzed RecA bound to ssDNA of various length (main length 580 to 800 nt) by electron microscopy (EM) and determined the size of nucleofilaments with oligonucleotides of 32 nt using mass photometry (MP). Figure 2A shows that ssDNA by itself did not yield substantial MP signals, while RecA and the 2 mutant versions each showed a well-defined peak around 80 kDa, consistent with the theoretical mass of 76 kDa for dimers. If monomers are populated in solution, they may not have been observable in our MP experiments, because their mass (38 kDa) is close to the detection limit of the instrument. Our MP measurements do not agree with our SEC experiments (Fig. 1A), in which we only observed monomers for all 3 variants. This is likely a result of the much higher salt concentrations used in the SEC experiment compared to the MP measurement (500 mM NaCl and 137 mM, respectively), wherefore we favor the view that under physiological conditions within cells, RecA forms dimers.

When incubated together with ssDNA, mass spectra of RecA showed 2 peaks, a well-defined peak at about 160 kDa, and a broad peak between 250 and 400 kDa (Fig. 2A). Both peaks are larger than dimers, indicating that they represent DNA- bound species. Using electron microscopy (EM), we could verify that RecA forms nucleoprotein (NP) filaments in the presence of ssDNA (Fig. 2B), but not in the absence of DNA (Fig. S1). NP filaments had a helical appearance to our eyes, in agreement with the helical nature of E. coli RecA filaments (22). These findings show that the C-terminal hexa-histidine tag did not strongly interfere with NP formation *in vitro*. We could not determine the exact stoichiometry implied by the peaks seen in MP experiments, because the measurements were calibrated with protein standards, which are inaccurate for DNA-containing complexes (40). We could not determine a pseudo- (protein calibrated) mass of free ssDNA oligos, because ssDNA did not yield appreciable scattering on its own, even when using poly-L-lysine coated slides. However, MP measurements of RecA clearly showed ssDNA binding at nanomolar concentrations, i.e., at physiologically relevant levels. For RecA_K70A_, we also observed DNA binding by MP and EM analyses, however revealing somewhat reduced formation of RecA-ssDNA filaments (Fig. 1A).

10.1128/msphere.00412-22.4 FIG S1 Electron microscopy of RecA variant protein with and without ssDNA. Electron micrographs show filament formation of wild type RecA and no filament formation in RecA mutants without ssDNA. Download FIG S1, TIF file, 1.5 MB. Copyright © 2022 Hernández-Tamayo et al. 2022 Hernández-Tamayo et al. https://creativecommons.org/licenses/by/4.0/ This content is distributed under the terms of the Creative Commons Attribution 4.0 International license.

MP spectra of the K70R mutant showed a single peak at around 80 kDa in the presence of ssDNA (Fig. 2A), consistent with free dimers, and only few polymeric structures were visible by EM. Interestingly, those corresponded to ring-shaped heptamers (with dimers not being well visible), indicating that there is a low propensity for the formation of polymers with a different architecture than wild type polymers (Fig. S1). We could not observe these heptamers in our mass photometry assays, likely because of the much lower concentrations used in MP experiments. In E. coli, RecA_K70R_ has virtually no ATP hydrolytic activity, but can bind ATP, and ssDNA (26). Thus, B. subtilis RecA lacking ATPase activity shows a strikingly different effect of no longer binding to ssDNA.

To directly test for binding to dsDNA, we performed electromobility shift (EMSA) assays. Figure 2C shows cooperative binding of wild type RecA to a 68 bp dsDNA were generated by annealing custom-made oligonucleotides. Both mutant versions of RecA showed cooperative DNA binding, but with lower affinity; complete binding was seen at 4 μM wild type RecA, but at 10 or 8 μM RecAK70A or RecAK70R, respectively.

These experiments show that, *in vitro*, B. subtilis RecA has a requirement of ATP binding activity for efficient binding to short DNA oligonucleotides and reveals that loss of ATPase activity additionally leads to the formation of aberrant nucleoprotein filaments, as judged from mass spectrometry and EM analyses. However, both mutations still allow for DNA binding to occur *in vitro*.

### Effects of Walker A mutations on the functionality of RecA.

We next moved our investigations to *in vivo* conditions. *RecA*_K70A_ and *recA*_K70R_ alleles were transferred into the chromosome of B. subtilis, under the control of the original *recA* promoter, generating a merodiploid strain in which the original *recA* gene is driven by the xylose promoter, and normally, is not induced during exponential growth. Figure 2A shows that all fusions were expressed as full length proteins, and no cleavage of mVenus occurs. Strikingly, while RecA can be expressed as a RecA-mVenus of RecA-sfGFP fusion as sole source of the protein (41, 42), we found that wild type RecA continues to be synthesized in the merodiploid strains expressing RecA-sfGFP even in the absence of xylose (Fig. 2B), suggesting that the sole presence of mutant *recA* alleles is detrimental for the cells, as is the gene deletion. As mentioned above, E. coli RecA_K70R_ is dysfunctional, and is has been shown to display a reduced SOS response activity compared to wild type RecA (43). Figure 2B shows that roughly equal amounts of wild type RecA and of fusion proteins were expressed. We tested for a defect in DNA repair via recombination by 2 approaches: (i) using an established system in which the HO endonuclease gene is integrated into the *amyE* locus on the chromosome, driven by the inducible xylose promoter, and the corresponding cut site being positioned close to the origin of replication, and (ii) using Mitomycin C (MMC). Expression of HO endonuclease was induced for a different length of time, followed by plating on plates lacking inducer, generating a DNA cut close to the origin regions of replication; this leads to cuts in about two-thirds of chromosomes in an exponentially growing B. subtilis culture (33, 44). As most cells contain separated origin regions for most of the cell cycle (45), some cells will contain no cut, some a single cut, and some cuts in both origin regions. Induction of HO endonuclease via xylose addition of addition of MMC increased levels of wild type and mutant RecA-sfGFP or wild type RecA-mV fusions, but not of wild type RecA, due to SOS induction of the *recA* but not of the xylose promoter (Fig. 2B). Wild type and mutant fusion proteins continued to be expressed at similar levels under induced conditions.

**FIG 3:**
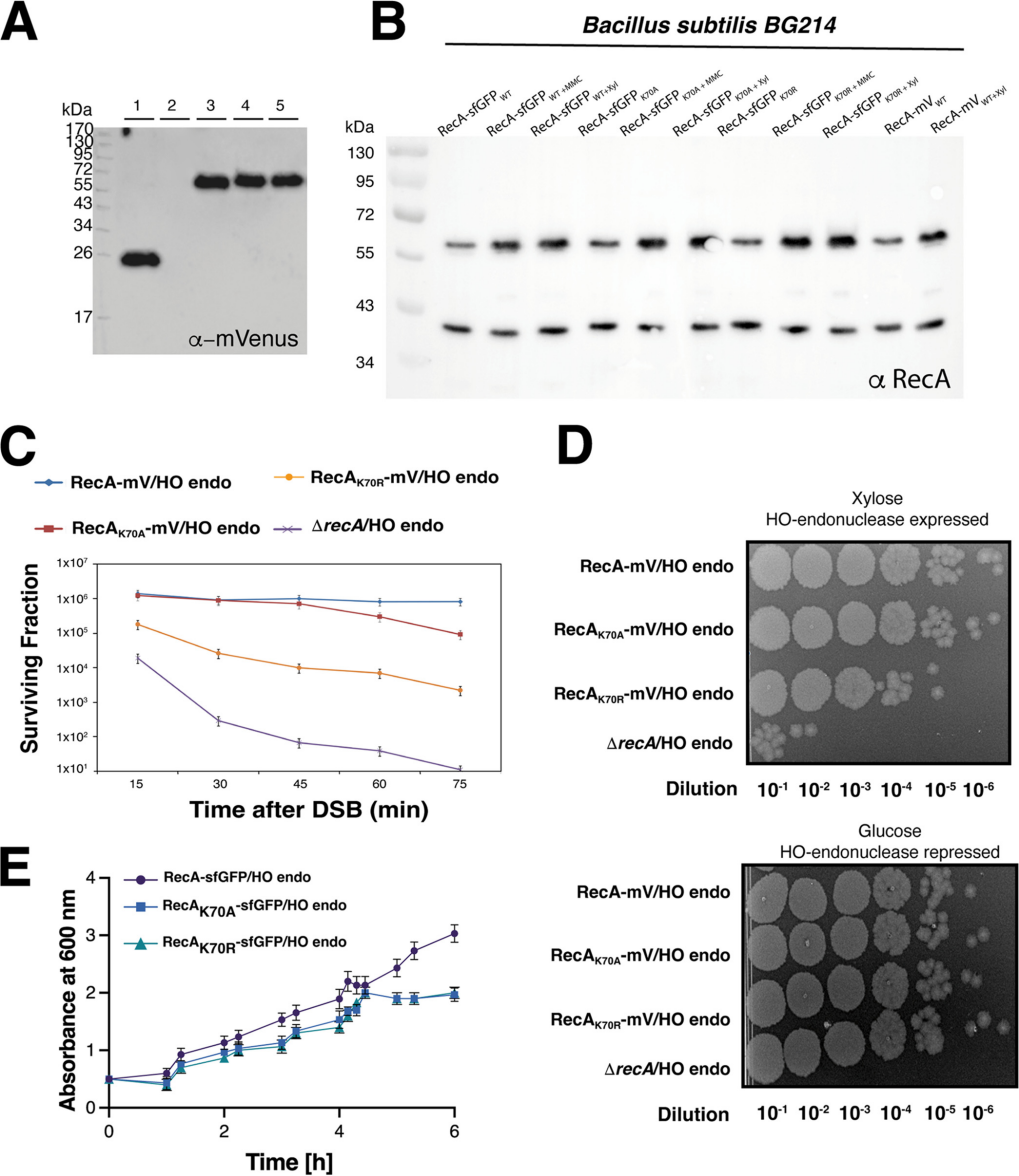
Survival assays and effects of RecA ATP-binding or ATPase mutants on the functionality of RecA. (A) Western blots of fluorescent protein fusions. Shown are Western blots from whole cell lysates of 1) E. coli expressing mVenus, 2) B. subtilis BG214, or 3) RecA-mV/HO endo, 4) RecA_K70A_-mV/HO endo and 5) RecA_K70R_-mV/HO endo expressing cells. Proteins were probed using a 1:500 dilution (rabbit-α-GFP) and secondary goat-α-rabbit-antibody (1:10000 dilution. Cells were harvested in exponential phase at OD_600_ 0.5 – 0.7 prior to analysis. (B) Shown are Western blots from whole cell lysates of cultures of BG214 strains containing *recA*_WT_-sfGFP *recA*_K70A_-sfGFP, *recA*_K70R_-sfGFP and *recA*_WT_-mV alleles expressing cells. Proteins were probed using a 1:500 dilution (α-RecA) and secondary antibody was added (goat-α-rabbit-antibody in 1:10000 dilution) after a series of washing steps with PBST. Cells were harvested in exponential phase at OD_600_ 0.5 – 0.7 prior to analysis. (C) Survival curves of strain RecA-mV/HO endo, RecA_K70A_-mV/HO endo, RecA_K70R_-mV/HO endo and Δ*recA*/HO endo, after induction of the DSB. All strains were plated in duplicate, in three independent experiments. (D) Spot assays. Cultures of RecA-mV/HO endo, RecA_K70A_-mV/HO endo, RecA_K70R_-mV/HO endo and Δ*recA*/HO endo, before and after DSB-induction. (E) Growth curves. Cultures of BG214 strains containing *recA*_WT_-sfGFP *recA*_K70A_-sfGFP and *recA*_K70R_-sfGFP alleles after treatment with MMC (30 nM), which was added at time point “0” in the growth curves.

In wild type cells, colony formation stayed roughly constant for the first 75 min after induction of HO (Fig. 3C). In the RecA_K70A_ strain, there was a minor loss of viability, while DSBs repair was strongly affected in the RecA_K70R_ strain. However, the latter retained some activity, in contrast to a *recA* mutant strain, which failed almost completely to deal with DSBs (Fig. 3C). The same effects were qualitatively found using spotting assays after HO endonuclease induction (Fig. 3D). Addition of a low dose of MMC to an exponentially growing culture (Fig. 3E, time point “0”) resulted in slower growth in both mutant strains compared with wild type cells.

These data show that reduced ATP binding activity and more so loss of ATPase activity has a dominant negative effect on RecA activity *in vivo*, affecting survival of cells by reducing DNA repair activity.

### ATPase activity is essential for the dynamics of RecA threads *in vivo*.

**FIG 4:**
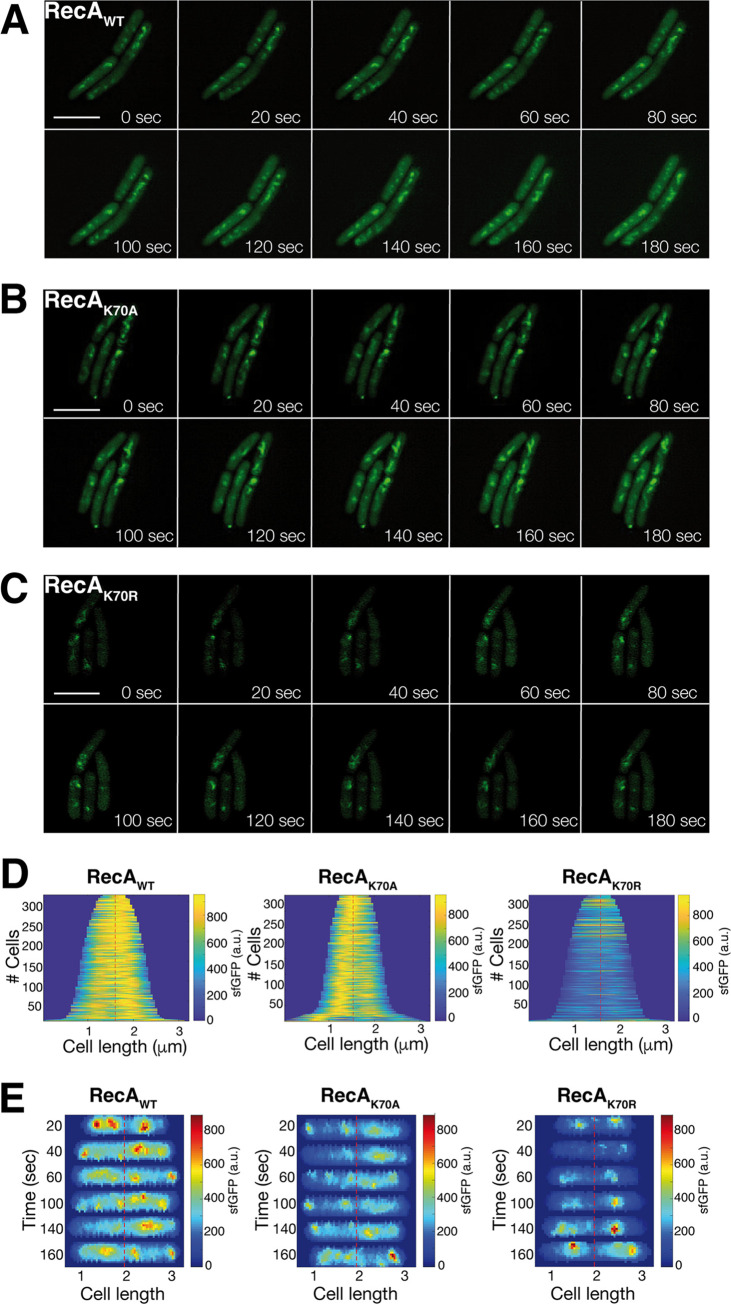
Structured illumination microscopy imaging of RecA thread formation in B. subtilis during double strand break repair. (A) to (C) Montage of movies (shown in supplementary material) with SIM reconstruction for RecA_WT_-sfGFP, RecA_K70A_-sfGFP and RecA_K70R_-sfGFP. Site specific DSBs were induced by the addition of 0.5% xylose for 30 min to exponentially growing cells. Z-stacks resulting from the sfGFP channel were merged and projected into tomographic representations. 20 ms acquisition time, scale bar 2 μm. (D) Demographs of RecA_WT_, RecA_K70A_ and RecA_K70R_ B. subtilis cells, showing the localization of RecA-sfGFP to the central regions. Cells were aligned and ordered according to size. The fluorescence profiles represent the mean fluorescence values along the medial axis after background subtraction and normalization such that the maximum fluorescence of each cell is equal. (E) Kymographs show examples of cells, in which signal intensities of RecA_WT_-sfGFP or of RecA_K70A_-sfGFP, but not of RecA_K70R_-sfGFP, frequently changed their subcellular positioning.

A major goal in this study was to investigate if the dynamics of RecA filaments depend on its ATPase cycle. GFP-RecA has been shown to first form a fluorescent focus at a site of a DSB, which then extends into thread-structures that remodel on a time scale of few minutes (33). In this study, we have used a C-terminal fluorescent protein fusion to RecA, which we have shown to fully complement for the wild-type protein *in vivo* (41, 42). This agrees with our finding that the C-terminus of RecA is permissive for the fusion of a histidine tag with regard to ssDNA binding *in vitro* (Fig. 2). We used structured illumination microscopy (SIM), a super resolution fluorescence microscopy technique, to obtain a better resolution of filamentous structures, and an even higher temporal resolution than earlier, acquiring images every 20 s rather than 60 s. Figure 4A shows that RecA threads changed in their subcellular arrangement between 20 s intervals (Movie S1). Foci or threads can appear between 20 s intervals and gain in fluorescence intensity. Threads frequently extend until they split into several entities, which can shift in their subcellular position. This behavior is very similar to what has been shown before using a lower temporal resolution. RecA dynamics were quite similar for RecA_K70A_, where we also observed fluorescent foci and threads of high fluorescence intensity (Fig. 4B and Movie S2). In striking contrast, RecA_K70R_ formed structures with much less intensity, and these did not show dynamics like the wild-type protein (Fig. 4C and Movie S3). To better visualize these finding, we generated demographics, in which signal intensities were plotted against cell length, showing that the alanine mutation slightly reduced signal intensities, while the arginine mutation drastically lowered focus/filament formation (Fig. 4D). Because expression of mutant and wild type RecA fusion proteins was similar (Fig. 3B), these data show that the formation of filaments is affected by arginine substitution, rather than by protein levels.

10.1128/msphere.00412-22.1 MOVIE S1 SIM reconstruction for RecA_WT_-sfGFP in B. subtilis. Cells were grown in the presence of xylose, such that RecA is expressed at physiological levels. Z-stacks resulting from sfGFP channel are merged and projected into tomographic representations. Fluorescent emissions are false colored in green. Movie Speed 12 fps. 20ms acquisition time, scale bar 2 μm. Download Movie S1, AVI file, 0.09 MB. Copyright © 2022 Hernández-Tamayo et al. 2022 Hernández-Tamayo et al. https://creativecommons.org/licenses/by/4.0/ This content is distributed under the terms of the Creative Commons Attribution 4.0 International license.

10.1128/msphere.00412-22.2 MOVIE S2 SIM reconstruction for RecA_K70A_-sfGFP in B. subtilis. Cells were grown in the presence of xylose, such that RecA_K70A_ is expressed at physiological levels. Z-stacks resulting from sfGFP channel are merged and projected into tomographic representations. Fluorescent emissions are false colored in green. Movie Speed 12 fps. 20ms acquisition time, scale bar 2 μm. Download Movie S2, AVI file, 0.07 MB. Copyright © 2022 Hernández-Tamayo et al. 2022 Hernández-Tamayo et al. https://creativecommons.org/licenses/by/4.0/ This content is distributed under the terms of the Creative Commons Attribution 4.0 International license.

10.1128/msphere.00412-22.3 MOVIE S3 SIM reconstruction for RecA_K70R_-sfGFP in B. subtilis. Cells were grown in the presence of xylose, such that RecA_K70R_ is expressed at physiological levels. Z-stacks resulting from sfGFP channel are merged and projected into tomographic representations. Fluorescent emissions are false coloured in green. Movie Speed 12 fps. 20ms acquisition time, scale bar 2 μm. Download Movie S3, AVI file, 0.1 MB. Copyright © 2022 Hernández-Tamayo et al. 2022 Hernández-Tamayo et al. https://creativecommons.org/licenses/by/4.0/ This content is distributed under the terms of the Creative Commons Attribution 4.0 International license.

Kymographs in Fig. 4E show examples of cells, in which signal intensities of wild type RecA frequently changed their subcellular positioning, while this was strongly reduced in ATPase mutant RecA. These finding reveal that loss of ATPase activity strongly reduces assembly of RecA into foci (i.e., assembling at DSBs) and into threads, while reduction of ATP binding has a noticeable but less pronounced effect on thread formation and their dynamics.

### *In vivo* dynamics of RecA at a single molecule level.

Dynamics of RecA have been visualized at single molecule level in *in vitro* experiments, but to our knowledge, there are no experiments of single molecule motion *in vivo*. We used a functional RecA-mVenus (“mV”) fusion (41, 42) to track single molecule dynamics during exponential growth, or after induction of DSBs. This was done using 20 ms stream acquisition, and we discarded tracks of less than 5 steps in order to avoid bias based on very short tracking events. Of note, we have shown that use of UV or blue light excitation slows down growth of B. subtilis cells or other bacteria, but not green light excitation (46), suggesting that use of 514 nm laser illumination does not induce harm to cells that might alter their physiological state.

**FIG 5:**
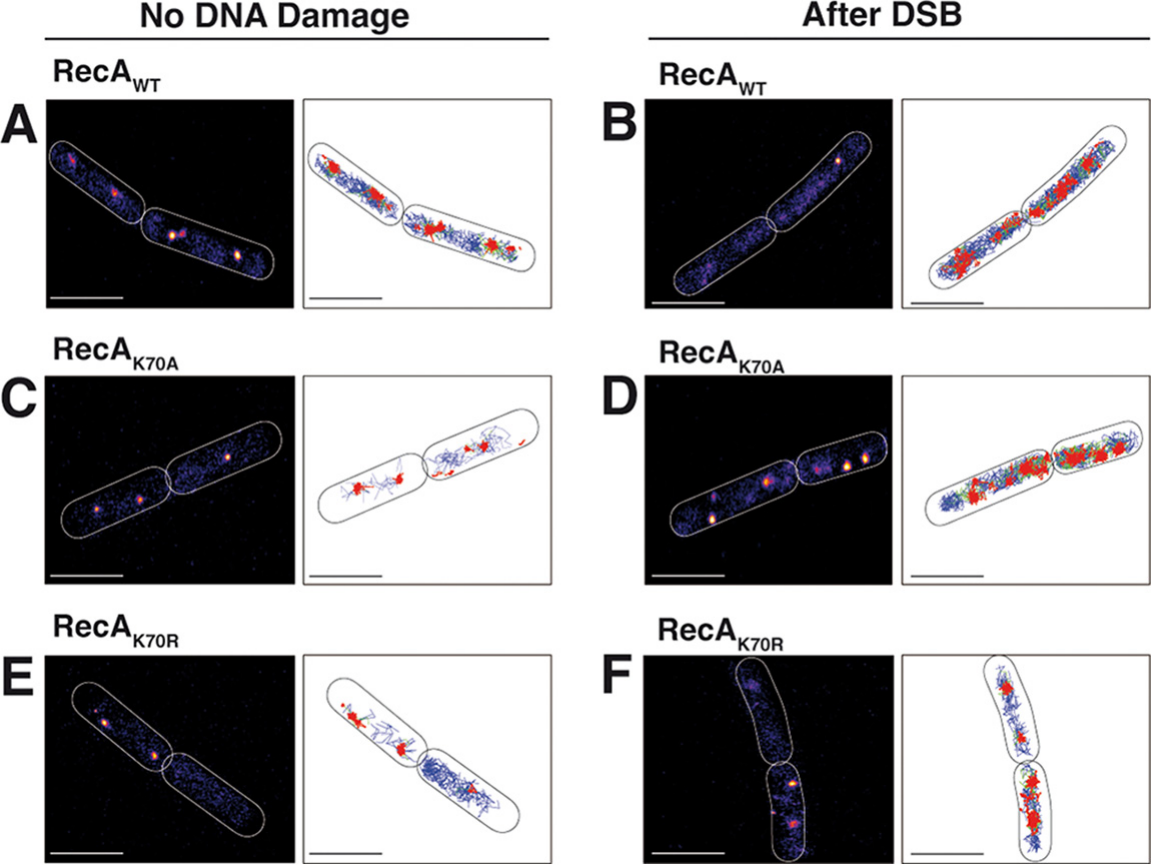
Overlay of all tracks (“Sum”) of single-molecule tracking movies of exponentially growing cells, expressing RecAwt-mV, RecAK70A-mV and RecAK70R-mV. Panels (A), (C), and (E) show cells with no DNA damage. Panels (B), (D), and (F) show cells after induction of a single DSB. Outlines of cells are indicated by white ovals, heat maps of localization on the left were generated using a background tool in the Fiji program plugin GDSC SMLS. Tracks shown in the right panels are generated in SMTraker program and show trajectories representing confined motion (red, staying within a circle of less than three times the localization error), molecules changing between confinement and free mobility (green, “mixed behavior”), and freely diffusive molecules (blue). White bars 2 μm.

Figure 5A shows an overlay of all frames of a typical movie (“sums,” left panel). RecA-mV can be seen to be concentrated in 2 or 3 subcellular regions. The right panel shows that these regions correspond to low mobility of molecules, as red tracks represent molecules showing very little displacement (less than three times the localization error) for at least 6 frames (“confined” motion). Blue tracks represent freely diffusing RecA-mV molecules, and these are mostly found in the central part of the cell, and not throughout the cell, e.g., like a freely diffusing enzyme (47). RecA has been shown to associate with replication forks after induction of DNA damage, e.g., via UV irradiation (48). Therefore, confined tracks likely represent RecA that is associated with replication forks even during exponential growth. Interestingly, when a single DSB is induced, RecA shows confined motion at many sites along the central part of the cell, likely representing RecA bound within the threads on the DNA seen by SIM experiments (Fig. 4). Confined motion at few sites within cells was also seen for the 2 RecA mutant proteins (Fig. 5C and E), an increased degree of confined motion could be clearly seen for the K70A mutant version (Fig. 5D). As will become apparent below, the K70R mutant behaved differently during DSB repair, which is not clearly visibly from single movies/sums images.

**FIG 6:**
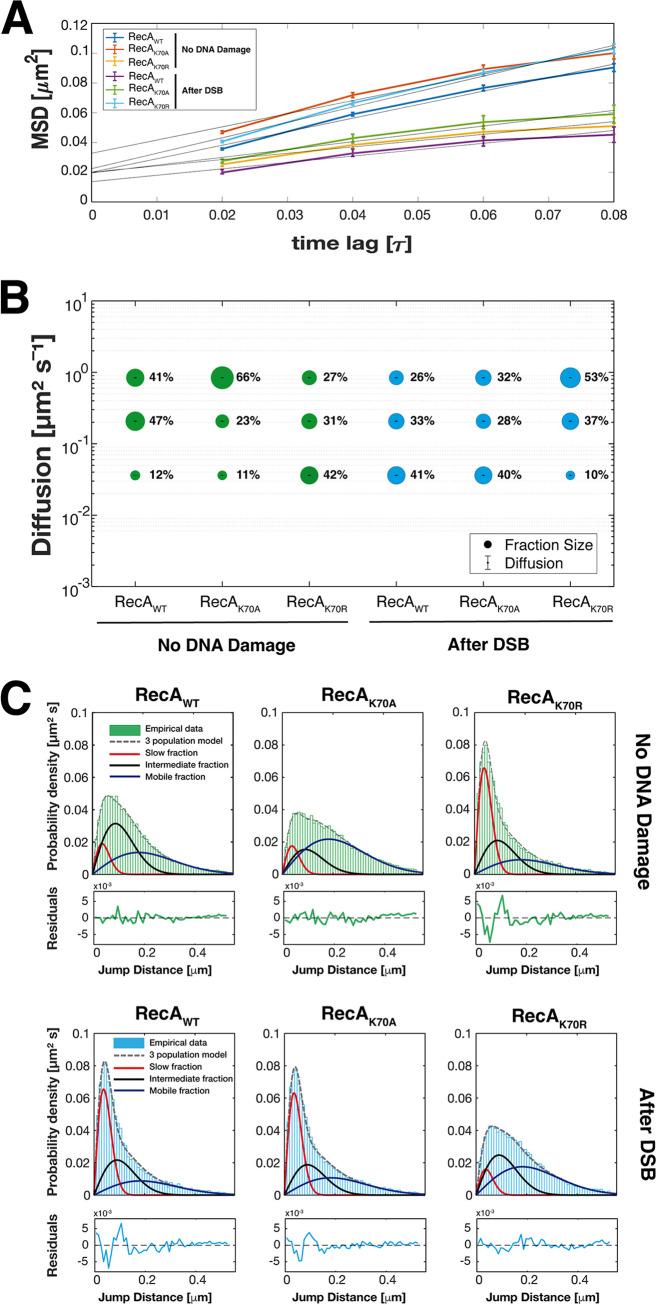
Analyses of single-molecule dynamics of RecA_WT_-mV, RecA_K70A_-mV and RecA_K70R_-mV expressed in B. subtilis cells during exponential growth. (A) Mean squared displacement (MSD) analyses of RecA_WT_-mV, RecA_K70A_-mV, and Reca_K70R_-mV, showing different overall diffusion constants. MSD assumes an overall diffusion regardless of sub-diffusion. (B) Bubble plot, derived from squared displacement analyses (SQD) shows the size of the fractions (proportional to the area) and corresponding diffusion coefficients in cells with no DNA damage and after DSB-induction. (C) Jump distance analysis shows probability of displacements, different colored solid lines represent the subpopulations; dotted lines represent the sum of the subpopulations.

Mean squared displacement analyses of all tracks obtained showed that wild type RecA and RecA_K70A_ had similar diffusion rates, while RecA_K70R_ moved much slower through the cells (Fig. 6A). After induction of a DSB via induction of HO endonuclease, RecA_WT_ showed high decreased mobility (Fig. 6A). This decrease was less pronounced for ATP binding-mutant RecA_K70A_, while ATPase mutant RecA_K70R_ showed the opposite behavior, with molecules becoming much more mobile. It must be kept in mind that the synthesis of wild type RecA is also induced in the strains expressing mutant RecA, when endonuclease is induced, such that the behavior of mutant RecA may be affected by the presence of some functional RecA molecules. To better interpret the drastic effects we observed, squared displacement analyses was employed to determine if monitored tracks might fall into different categories of mobility. The visualization of the results is shown in Fig. 6C in the form of jump distances, where the probability of different lengths of squared displacements (representing the number of certain lengths of motion done by molecules) is plotted. A single population of molecules would yield a single continuous probability distribution, or Rayleigh distribution. To explain all RecA tracks in a satisfactory manner, we fitted the data with 3 Rayleigh distributions (Fig. S2), resulting in an R^2^ value of 0.99. Using only 2 populations resulted in a stronger deviation of measured and modeled data, and 2 populations could not explain all jump distance events, as opposed to assuming 3 populations (Fig. S2). Using this fitting, we found a slow population of 12% (Fig. 6B, red curve in 5C), a medium-mobile population of 47% (Fig. 6C, black curve), and a high mobility fraction of 41% (Fig. 6C, blue, curve, Table 1). Taken together, the 3 Rayleigh distributions resulted in the fit indicated by the gray dotted line, which fits the data very well (Fig. S2).

**TABLE 1:** Diffusion constants of static, medium-mobile, and mobile molecule fractions

Strain	# cells	# tracks	D^*a*^	D_1_^*b*^	D_2_^*c*^	D_3_^*d*^
No Damage						
RecA_WT_-mV	138	4765	0.227 ± 0.018	0.036 ± 0.002	0.207 ± 0.001	0.840 ± 0.002
RecA_K70A_-mV	126	4623	0.221 ± 0.015	0.036 ± 0.001	0.207 ± 0.002	0.840 ± 0.001
RecA_K70R_-mV	132	4567	0.108 ± 0.017	0.036 ± 0.001	0.207 ± 0.001	0.840 ± 0.002
After DSB						
RecA_WT_-mV	131	4398	0.106 ± 0.011	0.036 ± 0.001	0.207 ± 0.002	0.840 ± 0.002
RecA_K70A_-mV	127	4800	0.132 ± 0.018	0.036 ± 0.002	0.207 ± 0.002	0.840 ± 0.001
RecA_K70R_-mV	133	5265	0.260 ± 0.027	0.036 ± 0.001	0.207 ± 0.001	0.840 ± 0.001

a *D*, MSD, average diffusion constant of all molecules (μm^2^·s^−1^).

b *D*_1_, diffusion constant of static fraction (μm^2^·s^−1^).

c *D*_2_, diffusion constant of medium-mobile fraction (μm^2^·s^−1^).

d *D*_3_, diffusion constant of mobile fraction (μm^2^·s^−1^).

10.1128/msphere.00412-22.5 FIG S2 Goodness of squared displacement analysis (SQD) fit and best model selection. Probability-probability plots for RecA and every mutant are displayed. In dark green, the model that performs better than the other one, -in light green- in terms of Mean Squared Error (MSE) and R-squared, where using a Triple-population fit clearly performs better, specially at the tails. Dotted lines indicate data predicted using random Brownian motion, solid lines indicate differences observed between modeled and measured data. On the highlighted area, details of the correlation and step-size histogram is shown along with the fit for both models. R-squared coefficients are higher than 0.998 in every of Triple-populations model case. “NT” = exponential growth, “DSB” = double strand break induced by HO endonuclease. Download FIG S2, TIF file, 0.8 MB. Copyright © 2022 Hernández-Tamayo et al. 2022 Hernández-Tamayo et al. https://creativecommons.org/licenses/by/4.0/ This content is distributed under the terms of the Creative Commons Attribution 4.0 International license.

The different states of mobility can be intuitively explained assuming freely diffusive RecA for the high-mobility population, RecA binding nonspecifically to dsDNA in the chromosome (medium mobility), and RecA being tightly bound ssDNA occurring in some cells due to spontaneously arising DNA damage. Figure 6C shows that track length for RecA_K70A_ is shifted to slightly larger values, resulting in an increase of likely freely diffusive RecA molecules at the expense of medium-mobile RecA. Strikingly, RecA_K70R_ mutant molecules showed strongly down-shifted track lengths, in agreement with the much lower MSD value (Fig. 6A). ATPase mutant RecA showed more than 40% of molecules in the slow-mobile state, at the expense of medium and fast-mobile molecules (Fig. 6B and C). Because lack of ATPase activity is expected to abolish RecA activity in active strand exchange and based on abnormal interaction of mutant RecA with ssDNA (Fig. 2B), we favor the view that mutant RecA_K70R_ forms nonproductive interactions with ssDNA and/or aggregates that lead to low mobility. In any event, our analyses show that loss of ATPase activity strongly affects RecA dynamics even during exponential growth.

### Loss of ATPase activity abolishes the response of RecA dynamics to the induction of double strand breaks.

After induction of HO endonuclease, inducing a single DSB (or 2, the system employed leads to cuts in about 75% of all chromosome loci [33]), population sizes for wild type RecA changed strongly, increasing 3.5 fold from 12% to 41%, while medium and fast-mobile fractions decreased accordingly (Fig. 6C). The visible formation of thread-structures (Fig. 4) supports the idea that the slow-mobile fraction of RecA molecules are those engaged in filament formation, suggesting that almost 50% of RecA is converted into its filamentous form. A very similar trend could be seen for ATP binding-mutant RecA, while ATPase mutant RecA showed completely opposing changes, in that the slow-mobile fraction became depleted, such that molecules had much higher mobility than before damage induction (Fig. 6B and C). Based on our assumption that the slow-mobile fraction refers to ssDNA-bound RecA, these experiments support the finding that ATPase mutant RecA fails to form dynamic filamentous structures *in vivo* (Fig. 4), and strongly malfunctions (Fig. 3).

**FIG 7:**
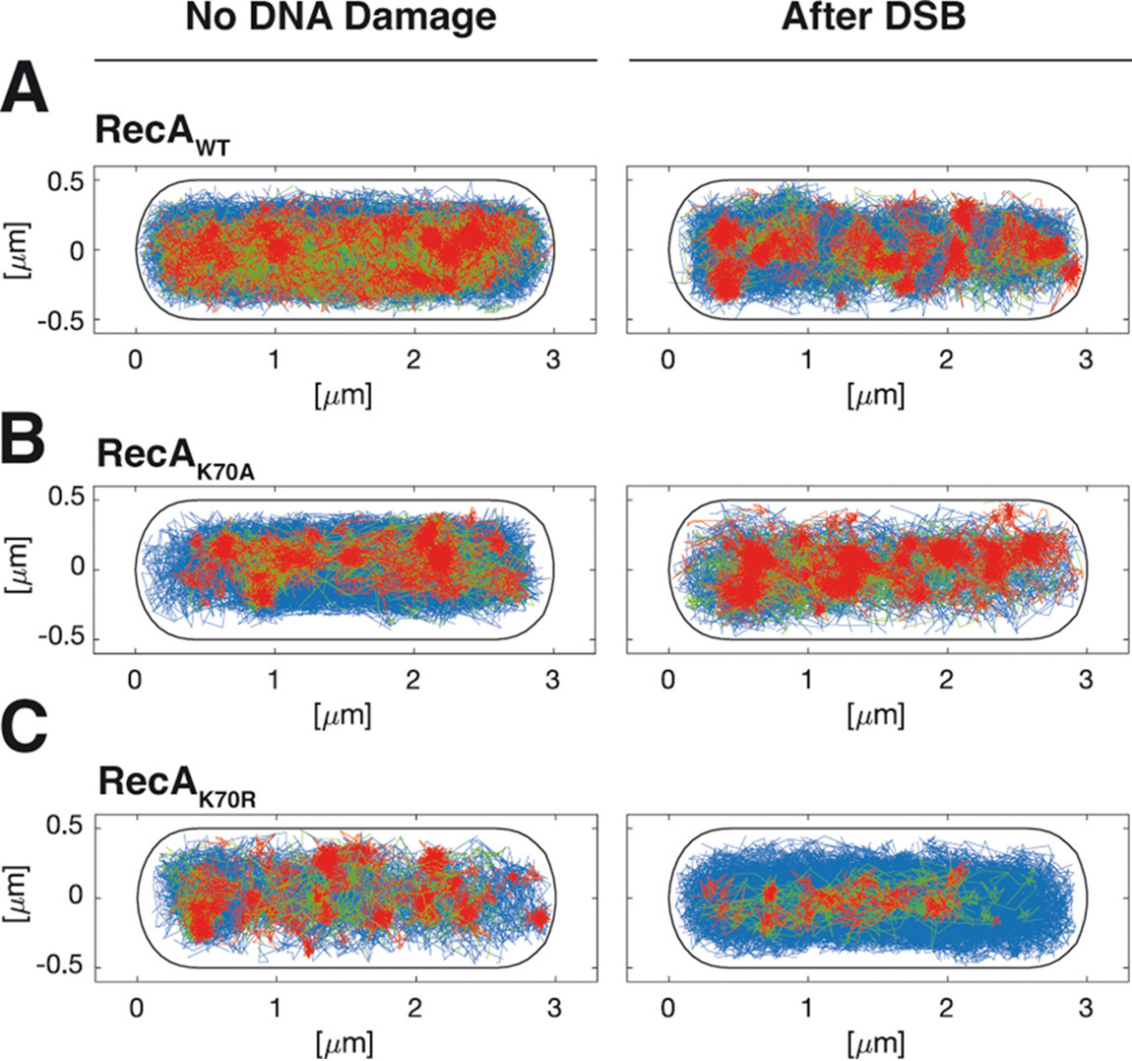
Confined motion of RecA molecules is restricted to nucleoid areas in the cell. (A) to (C) Confinement maps are an algorithm of SMTracker program, and are shown for RecA_WT_-mV, RecA_K70A_-mV, and RecA_K70R_-mV with no DNA damage and after induction of a DSB. A trajectory is considered to present confinement (red) when it has a dwell event (molecules staying within a radius of 120 nm) for at least 5 time points. Molecules changing between confinement and mobility are termed “transition” (mixed behavior), shown in green, and freely diffusive molecules lacking considerable parts of confinement are shown in blue.

In order to visualize changes in single molecule dynamics in 2D, all tracks were projected into a medium-sized cell of 3 × 1 μm (note that B. subtilis is about 0.8 μm wide), and were sorted into those that show very little motion for more than XY time intervals (“confined” motion, determined from three times the localization error), illustrated by red tracks in Fig. 7, those that show free diffusion (blue tracks), and those that show transitions between confined and free motion (green tracks, “transitions”). During exponential phase, wild type RecA reveals confined motion on the central part of cells containing the nucleoids, and free diffusion throughout cells (Fig. 7). After induction of DSBs, confined motion becomes more focused on the nucleoids, and transition events are visibly reduced, indicating that many molecules have been tightly incorporated into putative RecA/ssDNA filaments. RecA_K70A_ does not feature strong changes in the pattern of motion, the increase in confinement seen from SQD analyses is less visually apparent. Note that slow-mobile molecules will largely agree with molecules showing confined motion but are not identical with this fraction. Conversely, RecA_K70A_ revealed opposing changes, confinement was visibly highly reduced following induction of DSBs, while exponentially growing cells looks almost indistinguishable from wild type cells during DSB repair. Possibly, lack of ATPase activity leads mutant RecA stuck in filaments that cannot be depolymerized, such that infrequent repair events during growth fail to be resolved.

## DISCUSSION

RecA and Rad51 are the central players in homologous recombination (HR), which in turn is at the heart of repair of DNA damage. HR is essential for the repair double strand breaks arising in a chromosome, for transformation with foreign DNA, or for the restart of stalled replication forks (34, 49–51). While Rad51 can generally rely on spatially paired sister chromosomes during DSB repair in S-phase, bacterial cells segregate replicated chromosome regions soon after their duplication (with E. coli showing some delay), such that sister copies of duplicated chromosome sites are one or more microns apart (52–54). Therefore, during DSB repair occurring away from replication forks, RecA would need to scan thousands if not millions of base pairs within the chromosome(s) for a region that is homologous to that having a break. Interestingly, in E. coli and Caulobacter crescentus cells, broken sites are moved together, e.g., a cut at one origin region leads to a transient transversion of the other origin region through the entire cell (35) (in C. crescentus, origins are tethered to the cell poles) or in E. coli, into the cell center, where both origins meet (34, 36). As opposed to this, in B. subtilis, origin sites containing an inducible break site appear to stay within both cell halves during repair via HR (33). A hallmark for RecA activity is the formation of ssDNA-protein filaments *in vitro*, and the arising of filamentous structures *in vivo*, in response to the induction of DNA damage including generation of single DSBs, as well as of replication roadblocks, as has been shown using fluorescence microscopy (33, 34, 36, 37, 55). Filamentous forms appear to represent bundles of filaments (34, 36) that extend in a time frame of minutes, away from single, induced DSBs, to elongate along the length of rod-shaped cells. While clearly, these structures, termed “threads” in B. subtilis, are crucial intermediates during homology search within the chromosome(s), the mode of their dynamic remodeling has been unclear. Using 2 mutant forms of B. subtilis RecA, we show that in the presence of normal levels of wild type RecA protein, reduced ATP binding does not strongly alter RecA dynamics *in vivo*, but lack of ATPase activity strongly affects the formation of threads, as well as their dynamic remodeling.

For E. coli RecA, it has been proposed that filaments extending along the long axis of the cell search for homology at many sites along their entire length, as has been shown to function *in vitro* (56), to reduce a three-dimensional search for a homologous sequence toward a two-dimensional search. Our finding that ATPase activity is crucial for filament formation strongly supports this idea. We show that reduction of ATP binding (and secondary reduction of ATPase activity) has an only mild effect on thread formation by RecA, while loss of ATPase activity is detrimental for this process. Using fast super resolution microscopy analyses, we show that remodeling of RecA filaments occurs within 20 s intervals, and throughout the cell including nucleoids, rather than exclusively along the cell membrane, in agreement with an earlier study (36). At such a speed, RecA filaments could simultaneously test for homology within many chromosome segments; failure to identify sufficient homology would involve ATPase activity to also release RecA- covered ssDNA from duplex DNA, whereby filaments could either shrink when many RecA molecules are unbound and rebound (this can also occur *in vitro*, in the absence of new RecA “loaders”), or simply diffuse vertically along the short axis of the cell, such that eventually, the nucleoid is tested along its length as well as its depth. Three-dimensional diffusion is possible also for long polymers such as dsDNA itself, which shows considerable displacement within a time frame of seconds simply based on Brownian motion (57, 58).

**FIG 8:**
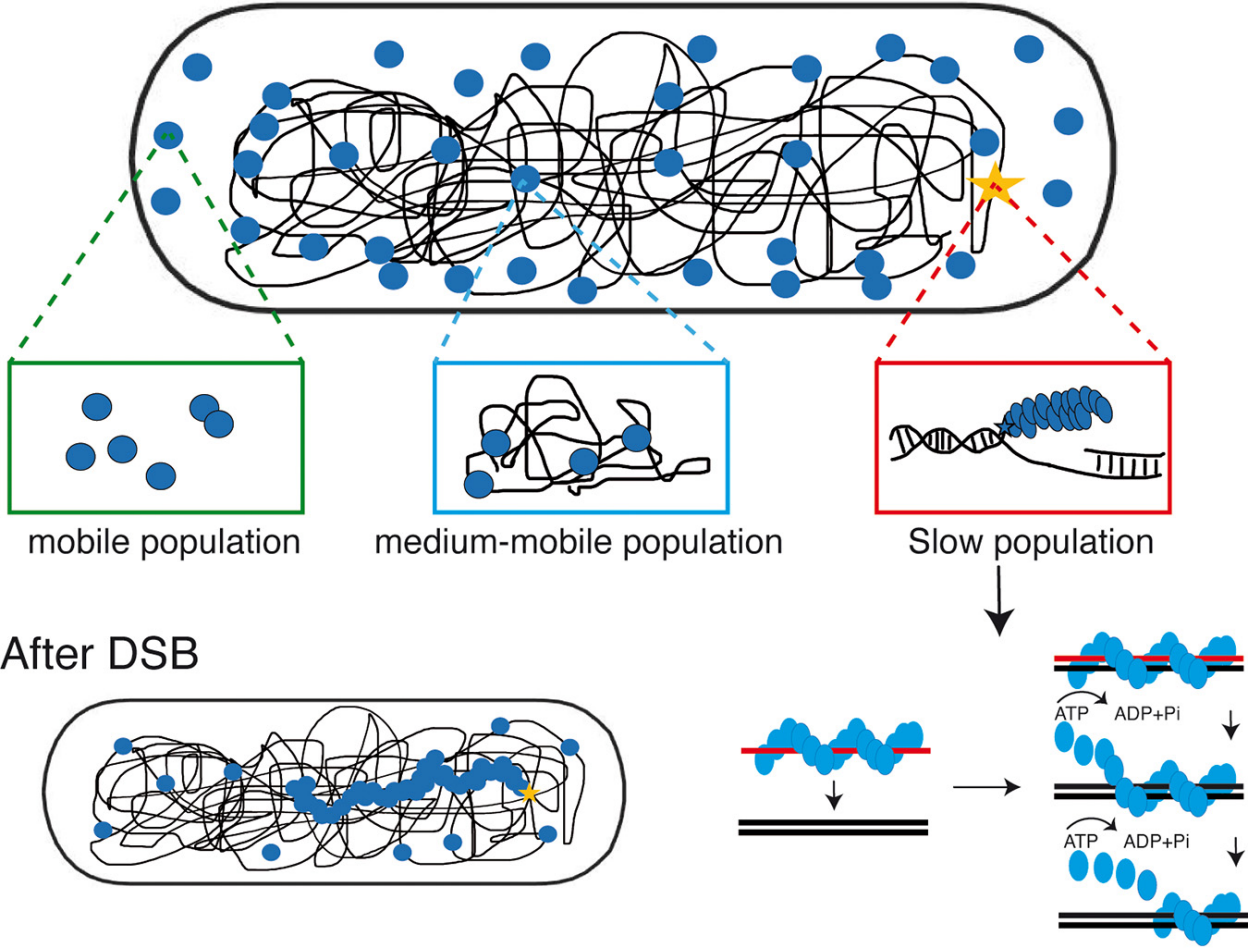
Model for the *in vivo* function of the ATPase-driven formation of dynamic RecA threads. RecA can bind either ssDNA or dsDNA, if it is recruited to ssDNA, e.g., at a site of a DNA break, it forms a helical filament. The helical ssDNA/RecA filament searches for a homologous region of dsDNA. Strand exchange then occurs, and the strand exchange process is driven by product stability, and factors such as RecG and RecU/RuvAB. RecA binding to undamaged dsDNA segments of the genome leads to constrained motion through the nucleoids, and filament formation is achieved by binding to ssDNA from freely mobile and from dsDNA-bound molecules.

We further extended our analyses using single molecule tracking of RecA. We found that trajectories collected from many cells could be well explained assuming three distinct (but interchangeable) populations: we observed (i) rapid diffusion throughout the cell (Fig. 7), likely representing freely diffusing RecA dimers (purified RecA forms predominantly dimers under physiological salt conditions) (Fig. 2A), (ii) medium-high diffusion, likely due to hopping between DNA strands based on nonspecific dsDNA binding by RecA, and (iii) low mobility, visible as confined motion on the nucleoids (Fig. 8). Low mobility is caused by RecA being bound within filamentous structures (Fig. 8), because the induction of a single DSB greatly increased this fraction, at the expense of medium – and high mobile fractions. Of note, most DNA binding proteins are largely present in constrained motion on the nucleoids, due to nonspecific DNA interactions (59), and indeed, a RecA-mV fusion largely localizes to the nucleoids using epifluorescence microscopy (33). Therefore, the 41% of RecA molecules that we determined may be an overestimate of free RecA diffusion, because the diffusion constant of 0.59 μm^2^ s^−1^ is quite low for a freely diffusing 128 kDa dimeric protein (RecA plus mVenus), suggesting that the high-mobility fraction also contains some (fast diffusing) molecules from the nonspecifically DNA-bound fraction. In any event, low mobility of 0.02 μm^2^ s^−1^ obtained in our analyses can only be explained by RecA molecules being part of a very large structure showing extremely low subcellular motion, such as filaments/threads observed in SIM microscopy (Fig. 4).

Our data revealing a strong reduction in medium – and fast-moving molecules to be recruited into RecA filaments is in agreement with earlier experiments showing that a redistribution of existing RecA molecules is sufficient for an efficient repair via RecA filaments (48). RecA having reduced ATP binding ability showed changes in single molecule dynamics like those of wild type RecA, in those 30 min after induction of a DSB, 40% of RecA molecules were in a low mobility/filament bound form, from 11% during exponential growth. Because RecA is a highly abundant protein (44), these data suggest that a massive number of RecA molecules is bound to ssDNA even if only a single DNA cut occurs within the genome. Contrarily, ATPase mutant RecA displayed completely aberrant behavior: during exponential growth, RecA_K70R_ showed a large, low mobility population, and less freely diffusive molecules, while induction of a DSBs freed molecules from the low mobility state. We interpret these findings to suggest that in the absence of ATPase activity, RecA cannot escape from nonproductive recombination events occurring during exponential growth. In other word, loss of ATPase activity appears to block disintegration of initial recombination events, such that RecA_K70R_ is erroneously often stuck in low mobility.

In E. coli, the intrinsic ATPase activity converts the high affinity binding form, RecA-ATP, to the low affinity binding form, RecA-ADP, driving dissociation from either the hybrid dsDNA products of strand exchange or from undamaged dsDNA. In eukaryotic cells, dissociation of Rad51 or Dmc1 from dsDNA requires the action of a RAD54 family dsDNA-specific translocase which uses the energy of ATP hydrolysis to dissociate the protein from strand exchange products or regions of undamaged dsDNA (24).

Surprisingly, purified ATPase mutant RecA was defective in nucleating nucleofilaments *in vitro*, in contrast to E. coli RecA (60). Because we did observe some filamentous RecA-mVenus structures *in vivo* following DSB induction, we argue that its defect appears to be partially overcome *in vivo*, likely because of the action of RecA loading or accessory factors such as RecO and RarA (41, 61). In any event, RecA from B. subtilis seems to differ in its molecular properties related to ssDNA binding compared with E. coli, so our study also strengthens the important idea that action of RecA at a molecular level, despite being generally conserved between organisms (including Rad51 from eukaryotes), must be viewed considering differing biochemical properties. This agrees with, e.g., RecA from the highly radiation resistant bacterium Deinococcus radiodurans operating in a different way during repair via HR than RecA in E. coli cells (62). Nevertheless, observations from this study clearly support the perception that the RecA nucleofilaments are tightly regulated by ATPase activity, including both ATP binding and ATP hydrolysis, to remove excessively assembled RecA on ssDNA. This tight regulation is critical to the success of HR *in vivo*.

In conclusion, our work reveals that ATPase mutant RecA retains only little, if any, activity of DNA repair via HR, and that dynamics of filaments/threads in cells is highly perturbed, suggesting that homology search along filaments is a major driver for directional search of sister regions for setting up Holliday junctions. Out data also agree with the idea of ATPase waves within RecA filaments generating a motor-like function proposed for E. coli cells (63) that could lead to spatial reorganization of filaments. Figure 8 shows a model in which continued ATPase activity with RecA filaments (only one is shown) leads to homology search along the long axis, to filament extension and diffusion toward the other end of the cell, made possible by the release of nonproductive strand exchange events.

While E. coli and C. crescentus cells set up HJs by joining the DSB site and the sister site (34–36), B. subtilis cells appear to do this across several micrometers, because break sites do not appear to move, while RecA threads can extend and retract (33). It will be intriguing to investigate how B. subtilis or possibly Gram-positive bacteria in general organize HJ formation in time and space.

## MATERIALS AND METHODS

### Bacterial strains and growth conditions.

The bacterial strains and plasmids used in this study are listed in Table S1, and the nucleotides are listed in Table S2. E. coli strain XL1-Blue (Stratagene) was used for the construction and propagation of plasmids and E. coli strain BL21 Star DE3 (Invitrogen) for the heterologous overexpression of proteins. All B. subtilis strains were derived from the wild type strain BG214. Cells were grown in Luria-Bertani (LB) rich medium at 37°C or 30°C. When needed, antibiotics were added at the following concentrations (in μg/mL): ampicillin, 100; chloramphenicol, 5; spectinomycin, 100; kanamycin, 30. When required, a filter-sterilized solution of xylose was added to media or agar plates to a final concentration of 0.5% xylose.

10.1128/msphere.00412-22.6 TABLE S1 Bacterial strains and plasmids. Download Table S1, DOCX file, 0.01 MB. Copyright © 2022 Hernández-Tamayo et al. 2022 Hernández-Tamayo et al. https://creativecommons.org/licenses/by/4.0/ This content is distributed under the terms of the Creative Commons Attribution 4.0 International license.

10.1128/msphere.00412-22.7 TABLE S2 Oligonucleotides used in this work. Download Table S2, DOCX file, 0.01 MB. Copyright © 2022 Hernández-Tamayo et al. 2022 Hernández-Tamayo et al. https://creativecommons.org/licenses/by/4.0/ This content is distributed under the terms of the Creative Commons Attribution 4.0 International license.

### Construction of strains.

RecA, RecA_K70A_, and RecA_K70R_ were visualized as a RecA-mV, RecA_K70A_-mV, RecA_K70R_-mV (for use in Single Molecule Microscopy) or RecA-sfGFP, RecA_K70A_ -sfGFP, RecA_K70R_ -sfGFP (with the goal to use in Structured Illumination Microscopy), fusion proteins expressed at the original locus. The entire *recA* variant ORF’s were integrated into vector pSG1164-mVenus or pSG1164-sfGFP, using ApaI and EcoRI restriction sites, and BG214 cells were transformed with the resulting constructs, selecting for cm resistance, leading to the strains in Table S1. This strategy resulted in merodiploid cells: one version was transcribed from the original *recA* promoter, the second was placed under the control of the xylose promoter and was not induced. We verified by sequencing that the point mutations were present in the expressed allele. For DSB studies, HO endonuclease system was integrated at *amyE* locus using the plasmid pSG1192, and expression was induced by xylose addition (0.5% final concentration) for 30 min (33, 37). Transformation of BG214 using was achieved by growing overnight cultures at 30°C and 250 rpm in liquid LB media (10 g sodium chloride per litter). The next day, we used a 200 mL shaking flask to inoculate 10 mL of freshly prepared liquid modified competence media (MCM) with our overnight culture to yield an optical density of 0.08–0.1 measured at 600 nm (OD_600_). MCM was prepared according to published procedures (64). The prepared culture was grown in MCM at 37°C under constant shaking (200 rpm) to ensure proper aeration till it reached stationary growth phase indicated by an OD_600_ of 1.4–1.6. For subsequent transformation of plasmid or chromosomal DNA, we used an aliquot of 1 mL from that culture and added a total of 1 μg of the respective DNA to it (either plasmid or chromosomal DNA); as a control, we used 1 mL of the same culture without any addition of DNA. Each culture was further incubated at 37°C in tubes with constant shaking for 2 more hours, followed by streaking out different amounts of culture aliquots onto fleshly prepared, solid LB-agar plates containing the appropriate antibiotics to maintain selective pressure for the respective strain.

For expression of soluble 6×His-RecA, 6×His-RecA_K70A_, and 6×His-RecA_K70R_ the coding sequence lacking the first 10 codons was amplified by PCR using chromosomal DNA from B. subtilis wild-type strain BG214. The fragment was further integrated in the expression vector pET28a (Novagen) by EcoRI and XhoI restriction ligation and brought into the expression host E. coli BL21(DE3) giving rise to the strains pET28a::*recA*HisTag, pET28a::*recA*_K70A_HisTag, and pET28a::*recA*_K70R_HisTag.

### Expression and purification of RecA variants.

Protein purification was performed in 2 consecutive steps. The purification of (His)6-RecA, RecA_K70A_, and RecA_K70R_ initially began with affinity chromatography using an ÄKTA Prime apparatus (GE Healthcare) and Nickel-Sepharose columns (HisTrap HP 1 mL, GE Healthcare) and was continued by size exclusion chromatography using an ÄKTA FPLC apparatus (GE Healthcare) and a gel filtration column (Superdex 75 16/60 GL, GE Healthcare). Prior to purification, the respective proteins were overexpressed in BL21 DE3 cells carrying a pET28a vector (Novagen) with an (indirectly) IPTG-inducible T7 promoter, 6 encoded histidine and the full gene sequence of the B. subtilis *recA* gene and *recA* variants. Transformants were grown under vigorous shaking in LB-medium at 37°C to exponential phase (OD_600_ 0.6) and induced for 60 min with 1 mM IPTG. Subsequently, the cells were centrifuged (20 min, 4°C, 5000 rpm) and the pellet was resuspended in HEPES A (50 mM HEPES, 300 mM NaCl, pH 7.5). To prevent protein degradation a protease inhibitor was added (Complete, Roche). Afterwards, the cells were French pressed (AMINCO French press, Laurier Research Instrumentation) in 2 consecutive cycles at approximately 20000 lb/in^2^, and the lysate was centrifuged (30 min, 4°C, 16000 rpm). The clear supernatant was passed through a filter (pore-size 0.45 μm, Filtropur S, Sarstedt) before injection into the loop of the ÄKTA Prime apparatus (preequilibrated with HEPES A and HEPES B [50 mM HEPES, 300 mM NaCl, 500 mM imidazole, pH 7.5]). The proteins were loaded onto the Nickel-Sepharose column, the column was washed with 20% HEPES B and the protein eluted with 100% HEPES B in fractions of 1 mL and checked by SDS-PAGE. Fractions containing significant amounts of the desired protein were assembled and loaded onto size exclusion chromatography columns (preequilibrated with HEPES A). The peak fractions were analyzed by SDS-PAGE and only pure protein fractions were assembled and stored at −80°C.

### Gel filtration.

Gel filtration (GF) of HisTag-RecA_WT_ and mutants after Nickel-Sepharose columns affinity chromatography continued by size exclusion chromatography using an ÄKTA FPLC apparatus (GE Healthcare) and a gel filtration column (Superdex 75 16/60 GL, GE Healthcare) standard size is shown with triangles in the upper part of the chromatogram. Fractions in panels showing SDS-PAGE correspond to elution fractions in GF fractions.

### ATPase assays.

RecA ATPase activity was measured using coupled spectrophotometric enzyme assay. The reaction was performed in 100 μL assay buffer (6 mmol/l MgCl_2_, 20 mmol/l KCl and 100 mmol/l Tris–HCl, pH 7.4) 1 mmol/l ATP and different concentrations of RecA (0–3.0 μM), RecA_K70A_ (0–3.0 μM), and RecA_K70R_ (0–3.0 μM), or vehicle (DMSO) and incubated at 37°C for 3 h. At the end of the incubation, the ATPase activity of RecA, RecA_K70A_, and RecA_K70R_ were assessed by malachite green reagent (Sigma-Aldrich) (0.0812% wt/vol malachite green, 2.32% wt/vol polyvinyl alcohol and 5.72% wt/vol ammonium molybdate in 6 mol/L HCl, and argon water mixed in a ratio of 2:1:1:2, vol/vol/vol/v). Reactions were analyzed in triplicate at an absorbance of 620 nm. The kinetic analysis of the RecA, RecA_K70A_, and RecA_K70R_ ATPase activity was carried out using a nonlinear regression fit of the experimental points to the Michaelis–Menten equation. Commercially available Salmon Sperm ssDNA was obtained from Sigma-Aldrich.

### Western blotting.

B. subtilis o*r* E. coli cultures (1 mL) were harvested by centrifugation. The pellet was resuspended in lysis buffer (20 mM Tris-HCl [pH 7.0], 10 mM EDTA, 1 mg mL^−1^ lysozyme, 10 g mL^−1^ DNase I, 100 g mL^−1^ RNase I, 1 tablet of Mini EDTA-free, EASY pack [Roche, protease inhibitor cocktail]), and incubated for 30 min at 37°C. Proteins were separated by running 12% sodium dodecyl sulfate-polyacrylamide gel electrophoresis (SDS-PAGE) and were transferred onto nitrocellulose membrane followed by blocking with 5% milk in PBST (80 mM Na_2_HPO_4_, 20 mM NaH_2_PO_4_, 100 mM NaCl, 0.2% [vol/vol] Tween 20). Proteins were probed using a 1:500 dilution (rabbit-α-GFP), 1:1000 dilution (rabbit-α-*recA*), or 1:1500 dilution (mouse-α-His) and secondary antibody was added (goat-α-rabbit-antibody in 1:10000 dilution or goat-α-mouse-antibody 1:10000) after a series of washing steps with PBST. Solution A (100 mM Tris pH 8.5, 2.5 mM Luminol, and 0.4 mM Coumaric acid) and Solution B (100 mM Tris pH 8.5, 0.02% [vol/vol] H_2_O_2_) were prepared and mixed followed by incubation for 2 min for chemiluminescence detection with ChemiDocTM MP System (BIO-RAD).

### Electromobility shift assays.

Electromobility shift assays (EMSA) were performed with increasing amounts (0 to 14 μM) of RecA_WT_, RecA_K70A_, and RecA_K70R_ and fragments of 68 bp dsDNA were generated by annealing custom-made oligonucleotides (5’ACACACACACACACACACACACACCCCCTTACACACACACACACACACACACACCCCCTTACCCCCTT -3′) (0.9 pmol, DNA-fragment, 68 bp). The reaction mixture with a final volume of 20 μL (6 mmol/l MgCl_2_, 20 mmol/l KCl, and 100 mmol/l Tris–HCl, pH 7.4, 1 mmol/l ATP) was incubated for 20 min at room temperature. Subsequently, the protein-DNA samples were mixed with 6xDNA loading buffer (30% glycerol [vol/vol], 300 mM boric acid, 300 mM Tris, 0.5 mg/mL bromophenol blue) and run on native poly-acrylamide gels (6%) in 50 mM boric acid and 50 mM Tris at a constant voltage (constant 200 V, 2 h, power source, VWR). Afterwards, the gel was placed in a beaker containing running buffer and DNA-stain (dilution 1:60000, GelRed nucleic acid gel stain, Biotium) and rotated for 20 min at room temperature prior to DNA-visualization by ultra- violet light (UV Transilluminator, UVP).

### Mass photometry.

Oligomeric states of proteins were determined on a One^MP^ mass photometer (Refeyn Ltd.). Microscope coverslips (1.5 H, 24 × 60 mm, Carl Roth) and CultureWell Reusable Gaskets (CW-50R-1.0, 3 × 1 mm, Grace Biolabs) were cleaned with 3 alternating rinsing steps of ddH_2_O and 100% Isopropanol, and dried under a stream of compressed air. Coverslips were coated with Poly-L-lysine by pipetting 7 μL solution (0.01%, Sigma-Aldrich) between 2 coverslips, incubation for 30 sec, dipping in and rinsing the coverslips with ddH_2_O after separation, and drying in an air stream. Silicone Gaskets with 4 cavities were adhered on coverslips. Prior to each measurement, 18 μL PBS (pH 7.4, RT) solution was pipetted into one cavity, and the instrument was focused. Two microliters of protein sample were added, mixed, and measured for 60 s at 100 frames per second using AcquireMP (Refeyn Ltd., v1.2.1). As a mass calibration, NativeMark Unstained Protein Standard (Thermo Fisher Scientific) was measured, and data was fit to a linear regression. Proteins were measured either alone or mixed with ssDNA oligonucleotides and 1 mmol/l ATP after incubation for 10 min at 37°C, and concentrations between 5 and 10 μM RecA_WT_, RecA_K70A_, and RecA_K70R_. All data was analyzed using DiscoverMP (Refeyn Ltd., v.1.2.3) (65, 66).

### Electron microscopy.

RecA filament formation was assayed by incubating 4 μM RecA, RecA_K70A_, and RecA_K70R_ in a buffer containing 25 mM Tris–HCl (pH 7.5), 10 mM MgCl_2_, ATP regeneration system (8 mM phosphocreatine, 10 U/mL creatine phosphokinase), 1 mM DTT, 3 mM ATP, 3% (vol/vol) glycerol, 7.5 mM NaCl, and 100 nM ssDNA (generated using primers in Table S2 or Salmon Sperm ssDNA) for 10 min at 37°C. Filaments were stabilized by addition of 3 mM ATPɣS and 3 min incubation at 37°C. The samples were spotted onto carbon coated grids (400 mesh) were hydrophilized by glow discharging (PELCO easiGlow). Five microliters of sample (protein, ATPɣS, MgCl_2_, ssDNA) and 1:5 dilutions of the assay were applied onto the hydrophilized grids, respectively, and negatively stained with 2% uranyl acetate after a short washing step with H_2_O_bidest_. Samples were analyzed with a JEOL JEM-2100 transmission electron microscope using an acceleration voltage of 120 kV. Images were acquired with a F214 FastScan CCD camera (TVIPS, Gauting).

### Single molecule microscopy and tracking.

Cells were spotted on coverslips (25 mm, Menzel) and covered using 1% agarose pads previously prepared with fresh S7_50_ minimal medium by sandwiching the agarose between 2 smaller coverslips (12 mm Marienfeld). All coverslips were cleaned before use by sonication in Hellmanex II solution (1% vol/vol) for 15 min followed by rinsing in distilled water and a second round of sonication in double distilled water. In contrast to the wide-field illumination used in conventional epifluorescence microscopy, the excitation laser beam used in our setup is directed to underfill the back aperture of the objective lens, generating a concentrated parallel illumination profile at the level of the sample, leading to a strong excitation followed by rapid bleaching of the fluorophores. When only a few unbleached molecules are present, their movement can be tracked. In addition, freshly synthesized and folded fluorophores become visible when the sample is excited again. When an observed molecule is bleached in a single step during the imaging, it is assumed to be a single molecule. Image acquisition was done continuously during laser excitation with the electron-multiplying CCD (EMCCD) camera iXon Ultra (Andor Technology). A total of 2,500 frames were taken per movie, with an exposure time of 20 ms (23 frames per second [fps]). The microscope used in the process was an Olympus IX71, with a ×100 objective (UAPON 100×OTIRF; numerical aperture [NA], 1.49; oil immersion). A 514-nm laser diode was used as excitation source, and the band corresponding to the fluorophore was filtered out. Of note, cells continued to grow after imaging, showing that there is little to no photodamage during imaging, while cells stop growing when exposed to blue light (below 480 nm). Acquired streams were loaded into Fiji ImageJ (67). Automated tracking of single molecules was done using the ImageJ plugin MtrackJ (68), or u-track 2.2.0 (69).

### Diffusion analysis of single molecule tracks.

Tracking analysis was done with u-track-2.2.0 (69), which was specifically written for Matlab (MathWorks, Natick). Only trajectories consisting of a minimum of 5 frames were considered tracks and included for further analysis. A widely accepted method to analyze the diffusive behavior of molecules is by using the mean squared displacement (MSD)-versus-time-lag curve. This provides an estimate of the diffusion coefficient as well as of the kind of motion, e.g., diffusive, sub-diffusive, or directed. However, the method requires that, within a complete trajectory, there is only one type of homogeneous motion and that the trajectory is preferably of infinite length. To distinguish immobile and mobile molecules from each other, we compared the frame-to-frame displacement of all molecules in x and the y directions. Using a Gaussian mixture model (GMM) to fit the probability density distribution function of all frame-to-frame displacements, determine the standard deviations σ_1_, σ_2_ and σ_3_, as well as the percentages F_1_, F_2_, and F_3_ of the static, medium-mobile, and the mobile subfractions of molecules, respectively. Finally, the diffusion constants were calculated accordingly: $D_{i}=\frac{\sigma^{2}}{2\Delta t},\;\left(i=1,2\right)$, where *Δt* is the time interval between subsequent imaging frames. Generation of trajectories maps and visualization of static, medium-mobile, and the mobile tracks in a standardized cell are based on a custom written Matlab script (SMTracker 2.0) that is available on request (70).

### Structured illumination microscopy.

Samples at mid-exponential phase were mounted on ultrapure-agarose slides dissolved in LB (1%) for immobilization of cells prior to image acquisition. For localization experiments, image Z-stacks (~100 nm steps) were acquired using brightfield (BF) image acquisition (transmitted light) or structured illumination microscopy (SIM) with a ZEISS ELYRA PS.1 setup (Andor EMCCD camera, 160 nm pixel size; 3× rotations and 5× phases per z-slice; grating period: 42 μm; 100 mW laser line [between 80 and 200 W/cm^2^] at excitation laser wavelength 488 nm; ZEISS alpha Plan-Apochromat 100x/NA 1.46 Oil DIC M27 objective). SIM reconstructions were processed using ZEN-Black software by ZEISS. ImageJ2/Fiji version 1.52p was used for visualization and image processing (67, 71–73). Region(s) of interest (ROI) were defined by cell borders using the brush-selection tool to maintain good contrast levels of cellular areas. SIM reconstructions were manually cropped in axial and lateral dimensions, depending on plausibility of cellular positions, using the “Duplicate”-function. Signal located outside cell borders was background and was therefore eliminated. Resulting image z-stacks were projected using the Fiji implemented “Z-project”-function (e.g., “Average Intensity”), false-colored, and color-balance adjusted to generate tomographic representations. Three-dimensional SIM image z-stacks movies were visualized using the Fiji implemented 3D-Project function (with interpolation) for 360° visualization and z-stacks for a tomographic walk-through. Resulting 3D-visualizations were generated with merged channels, processed, and transformed as.avi movies, and finally combined in a sequential manner using Fiji.
