# Supplementary figures and images for: ATPase Activity of Bacillus subtilis RecA Affects the Dynamic Formation of RecA Filaments at DNA Double Strand Breaks
Source: mSphere. 2022 Nov 2;7(6):e00412-22. doi: 10.1128/msphere.00412-22 (PMC9769622; doi:10.1128/msphere.00412-22)

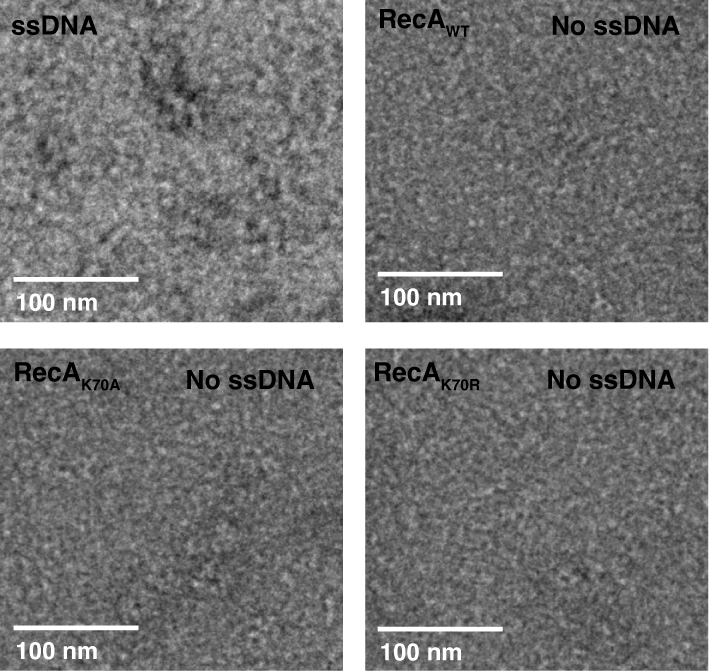

Supplement: FIG S1 [file msphere.00412-22-s004.tif]

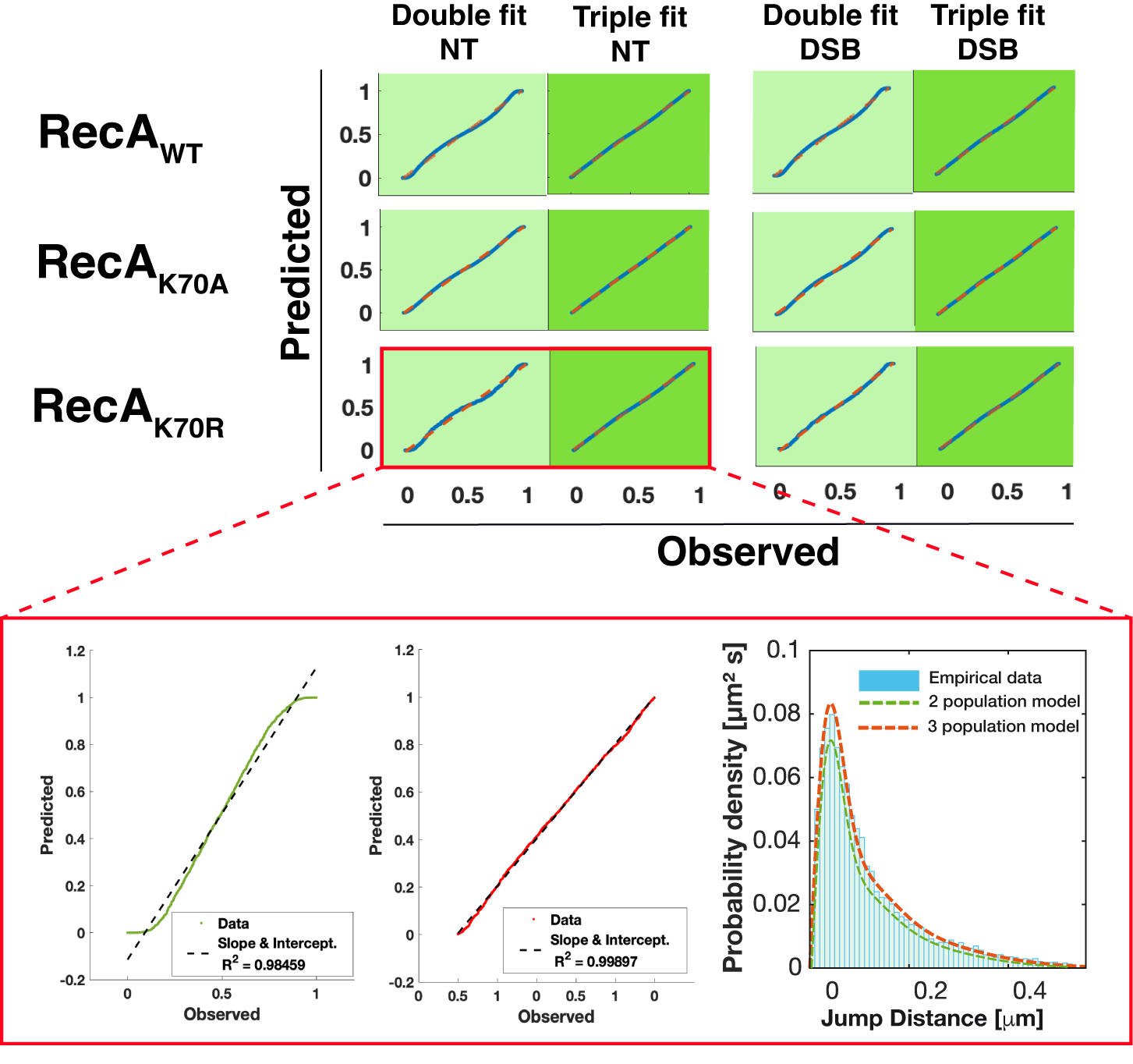

Supplement: FIG S2 [file msphere.00412-22-s005.tif]
