# Supplementary material for: ATPase Activity of Bacillus subtilis RecA Affects the Dynamic Formation of RecA Filaments at DNA Double Strand Breaks
Source: mSphere. 2022 Nov 2;7(6):e00412-22. doi: 10.1128/msphere.00412-22 (PMC9769622; doi:10.1128/msphere.00412-22)
Supplement: TABLE S1 [file msphere.00412-22-s006.docx]

**TABLE S1** Bacterial Strains and plasmids.

| Strain or Plasmid | Relevant features | | Reference or source |  |
| --- | --- | --- | --- | --- |
| *B. subtilis* |  |  | | |
| BG214 | Wild type |  | | |
| BG190 | Δ*recA recA::cat* | Alonso et al., 1991 | | |
| DK52 | *pxyl–HO* endonucleas*e::amy -HOcut at spo0J (359°C)* | Kidane et al., 2005 | | |
| PG7000 | pSG1164::recA_WT_-mVenus^cmR^ / *HO* endo | This study | | |
| PG7001 | pSG1164::recA_K70A_-mVenus^cmR^/ *HO* endo | This study | | |
| PG7002 | pSG1164::recA_K70R_-mVenus^cmR^/ *HO* endo | This study | | |
| PG7003 | pSG1164::recA_WT_-sfGFP^cmR^/ *HO* endo | This study | | |
| PG7004 | pSG1164::recA_K70A_-sfGFP^cmR^/ *HO* endo | This study | | |
| PG7005 | pSG1164::recA_K70R_-sfGFP^cmR^/ *HO* endo | This study | | |
| *E. coli* |  |  | | |
| DH5α | *sup*E44 Δ*lac*U169 φ80d*lac*ZΔM15 *hsd*R171 *recA*1 *endA*1 *gyrA*96 *thi-*1 *relA1* | New England Biolabs  (NEB) | | |
| BL21  (DE3) | *fhu*A2 [lon] *omp*T gal (λ DE3) [dcm] Δ*hsd*S | New England Biolabs  (NEB) | | |
| pET28a | DH5α pET28a HisTag fusion and expression vector, Kan^R^ | Novagen | | |
| PG8000 | DH5α pSG1164::recA_WT_-mVenus^cmR^ | This study | | |
| PG8001 | DH5α pSG1164::recA_K70A_-mVenus^cmR^ | This study | | |
| PG8002 | DH5α pSG1164::recA_K70R_-mVenus^cmR^ | This study | | |
| PG8003 | DH5α pSG1164::recA_WT_-sfGFP^cmR^ | This study | | |
| PG8004 | DH5α pSG1164::recA_K70A_-sfGFP^cmR^ | This study | | |
| PG8005 | DH5α pSG1164::recA_K70R-_sfGFP^cmR^ | This study | | |
| PG8006 | BL21 (DE3) pET28a:: recA_WT_-HisTag ^KanR^ | This study | | |
| PG8007 | BL21 (DE3) pET28a:: recA_K70A_-HisTag ^KanR^ | This study | | |
| PG8008 | BL21 (DE3) pET28a:: recA_K70R_-HisTag ^KanR^ | This study | | |
|  |  |  | | |
