# Supplementary material for: ATPase Activity of Bacillus subtilis RecA Affects the Dynamic Formation of RecA Filaments at DNA Double Strand Breaks
Source: mSphere. 2022 Nov 2;7(6):e00412-22. doi: 10.1128/msphere.00412-22 (PMC9769622; doi:10.1128/msphere.00412-22)
Supplement: TABLE S2 [file msphere.00412-22-s007.docx]

**TABLE S2** Oligonucleotides used in this work.

| Name | Sequence^a,b^ |
| --- | --- |
| RecAamydw310 | *TAT*GAATTCACCCCCTTCTTCAAATTCGAGTTCTTCTTG |
| RecAamyup315 | *GCA*GGGCCCATGAGTGATCGTCAGGCAGC |
| Hoendoup304 | *CA*GGGCCCAGGAGGTTACCGAATGCTTTCTGAAAACACGAC |
| Hoendodw305 | *TAT*GAATTCTTAGCAGATGCGCGCACC |
| mRFPup | *CAG*GGTACCATGGCCTCCTCCGAGGAC |
| mRFPdw | *TAC*GGGCCCCCCCACCGGGCGCCGGTGGAGTG |
| RecAORFdw | *AA*GGGCCCTTCTTCAAATTCGAGTTCTTCTTGTG |
| RecAORFup | *AC*GAATTCATGAGTGATCGTCAGGCAGCCTTAGATAT |

^a^ Non-encoded bases introduced as clamps are shown in italics. Restriction sites are underlined.

^b^ The location is indicated by the first 5’ nucleotide and the replicon where the sequence is located. Accession numbers are *recA* (CP053102.1 (1764677 to 1765723) of *B. subtilis.*
